# Single-organelle DNA-sequencing of chloroplasts and mitochondria in *Arabidopsis thaliana*

**DOI:** 10.1186/s12870-026-08232-3

**Published:** 2026-01-26

**Authors:** Zikai Xiang, Kazuki Takahashi, Chang Zhou, Hideki Takanashi, Shin-ichi Arimura, Masahito Hosokawa

**Affiliations:** 1 Waseda Research Institute for Science and Engineering, Waseda University, 3-4-1 Okubo, Shinjuku-Ku, Tokyo, 169-8555 Japan; 2 Research Organization for Nano and Life Innovation, Waseda University, 513 Wasedatsurumaki-Cho, Shinjuku-Ku, Tokyo, 162-0041 Japan; 3 Microbe Division/Japan Collection of Microorganisms, RIKEN BioResource Research Center, Tsukuba, Ibaraki 305-0074 Japan; 4 Graduate School of Agricultural and Life Sciences, The University of Tokyo, 1-1-1 Yayoi, Bunkyo-Ku, Tokyo, 113-8657 Japan; 5 Graduate School of Advanced Science and Engineering, Waseda University, 2-2 Wakamatsu-Cho, Shinjuku-Ku, Tokyo, 162-8480 Japan

**Keywords:** Single-organelle sequencing, *Arabidopsis thaliana*, Chloroplast, Mitochondria

## Abstract

Organelle genomes govern bioenergetics and stress responses, yet bulk sequencing masks heterogeneity. We establish single-organelle DNA-sequencing by adapting a droplet-based workflow to mitochondria and chloroplasts, using a sorbitol-based isolation that preserves organelles for encapsulation, amplification, and sorting. In *Arabidopsis thaliana* leaves, 313 of 384 single-organelle amplified genome libraries passed quality control (261 mitochondrial, 52 chloroplast). Genome coverage breadth at 1000 × sequencing depth showed medians of 59.5% and 84.5%, with maxima of 97.2% and 100%. Read-depth profiles were more fragmented for mitochondria and more uniform for chloroplasts, consistent with mitochondrial heterogeneity. This workflow enables analyses of recombination, rearrangements, and heteroplasmy.

**Supplementary Information:**

The online version contains supplementary material available at 10.1186/s12870-026-08232-3.

## Background

Chloroplasts and mitochondria are essential organelles in plant cells, supporting photosynthesis and respiration while retaining their own genomes (chloroplast DNA, cpDNA; mitochondrial DNA, mtDNA) that function in concert with the nuclear genome [1–4]. Plant mitochondria and chloroplasts carry genomes whose copy number, physical organization, and sequence composition vary across tissues and environmental conditions, influencing development, stress responses, and crop traits [5–11]. To date, conventional “bulk” organelle sequencing averages across many molecules [12–14] and has enabled high-quality genome assemblies for various plant species using short-read and long-read sequencing platforms [15–17]. For example, a survey of 1,531 *Arabidopsis thaliana* accessions revealed > 5,000 chloroplast and 1,400 mitochondrial variants, reflecting organellar population structure [18]. These data provide references for cross-accession and interspecies comparisons. However, bulk methods obscure heterogeneity among individual organelles by masking uneven genomic representation [19, 20], rearranged isoforms [21, 22], and low-frequency variants that may be functionally relevant [23, 24].

Single-cell genomics has shown that physical isolation coupled with whole-genome amplification can reveal hidden variation in complex systems, and this approach has been applied across organisms ranging from bacteria to mammalian tissues [25–29]. However, plant organelles remain underexplored at the single-organelle level [30]. A method that delivers per-organelle genome readouts at scale would provide direct measurements of reconstruction breadth and genome representation evenness, enable comparisons between mitochondria and chloroplasts, and establish a baseline for studying heteroplasmy, recombination, and genome maintenance across tissues and conditions [18, 31]. In this study, we describe such a workflow based on droplet encapsulation and whole-genome amplification tailored to plant organelles, and we outline quantitative criteria to evaluate single-organelle datasets.

## Results

### Single-organelle DNA sequencing workflow using the SAG-gel platform

**Fig. 1:**
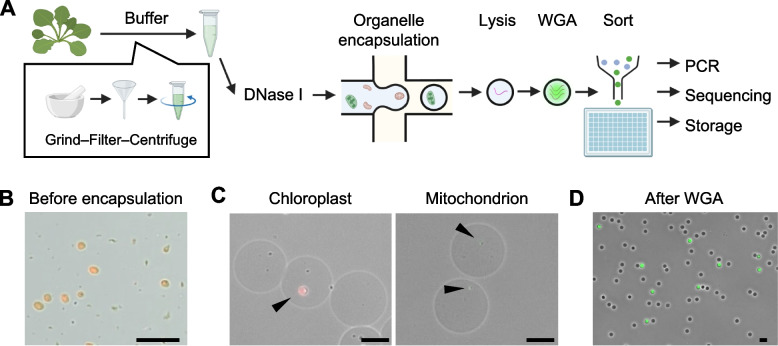
Single-organelle DNA-sequencing workflow. **A** Workflow schematic. Organelle suspensions are prepared from leaves of *A. thaliana* Col-0 in a sorbitol-based buffer that preserves intact mitochondria and chloroplasts, while depleting free DNA by DNase. DNase was inhibited with EDTA, followed by washing with buffer. Suspensions are encapsulated at limiting dilution into agarose gel droplets, followed by lysis, whole-genome amplification (WGA) by multiple displacement amplification (MDA), fluorescence-based identification of amplified agarose gel beads, sorting into plates, and library construction. **B** Representative field of the crude preparation. Intact chloroplast morphology was largely preserved (chlorophyll autofluorescence), with few ruptured organelles and fragments. Scale bar: 30 µm. **C** Representative micrographs of encapsulated organelles. Discrete chloroplasts (chlorophyll autofluorescence) and mitochondria (Rhodamine 123) are observed within agarose beads before lysis and genome-amplification. Scale bars: 15 µm. **D** Representative micrograph of amplified organelle genomes. Microscopy image of gel beads after WGA, stained with SYBR Green. SYBR green-positive beads indicate successful WGA of encapsulated DNA. Scale bar: 50 µm

We implemented a droplet-based single-organelle DNA-sequencing workflow previously named as single amplified genome (SAG)-gel [25, 26] in *A. thaliana* leaves. Organelle suspensions were prepared in a sorbitol-based buffer to preserve intact particles while depleting free DNA by DNase I [32–34] (Fig. 1A-B, Additional file 1: Figure S1−2), then randomly encapsulated at limiting dilution in agarose droplets so that most droplets contained either zero or one organelle (Fig. 1C). After droplet solidification, organelles were lysed and their DNA was amplified by multiple displacement amplification (MDA) within the agarose gel beads [25–28]. Upon SYBR Green staining, fluorescence gel beads (Fig. 1D) were identified successful genome amplification events for flowcytometric sorting of individual gel beads into well-plates for subsequent sequencing library construction. The percentage (%) of gel beads scored as positive was 7.3 ± 1.3 (mean ± SD), providing sufficient input for downstream analyses.

Physically isolating individual organelles, including mitochondria and chloroplasts, in agarose droplets and amplifying their genomes in parallel enables large-scale, per-organelle sequencing with standard library preparation formats, and it provides a clear basis for quantitative single-organelle evaluation. Droplet encapsulation with fluorescence-guided sorting sustains high throughput while preserving physical separation of templates. Limiting dilution keeps multi-occupancy rare, and a DNase-treated, organelle-preserving sorbitol isolation reduces free DNA and nuclear carryover without harsh treatments, which in turn allows concurrent recovery of mitochondrial and chloroplast libraries from the same preparation. Rigorous sample pretreatment is critical. If organelle fractions are not highly purified, carryover from the host nuclear genome, fragmentation of organelle DNA, and loss of intact organelles can markedly degrade data quality (Additional file 1: Figure S3-4). The optimized workflow supports high-throughput single-organelle genome sequencing with minimal nuclear carryover and preserved organelle integrity, enabling quantitative analyses of individual organelle genomes at scale.

### Quality metrics of single-organelle sequencing

After the sequencing of pooled library, quality control excluded SAGs dominated by nuclear carryover by aligning reads to a nuclear reference in which nuclear-mitochondrial DNA segments (NUMTs) were masked [35], thereby limiting false assignment of mitochondrial signal. DNA based quantitative PCR (qPCR) of organelle preparations showed that DNase I treatment reduced nuclear *cyo1* signal to near the detection limit while two mitochondrial loci outside the NUMT region (*cox1* and *rrn26*) remained readily detectable (Additional file 1: Figure S5), indicating strong depletion of nuclear DNA and supporting that residual NUMTs make only a minor contribution to mitochondrial read assignment.

**Fig. 2:**
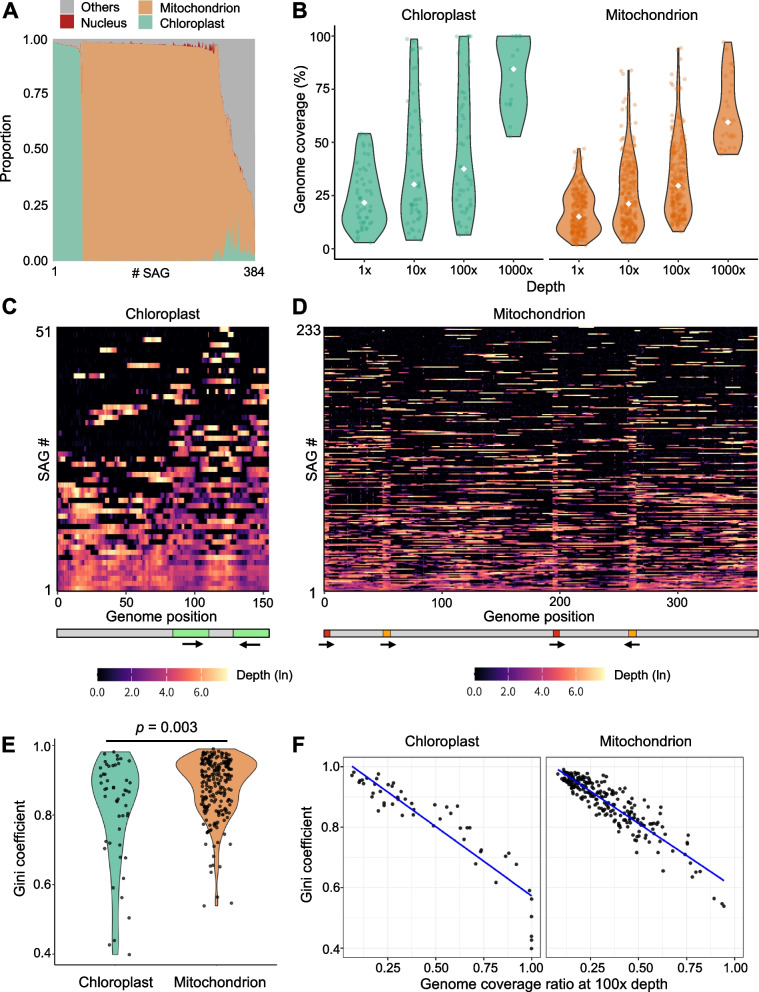
Composition, genome coverage, and coverage evenness of single-organelle genome sequencing of chloroplasts and mitochondria in *Arabidopsis thaliana*. **A** Library composition and quality control. Bar charts show sequencing read assignment proportions of the SAGs obtained in this study. Mitochondrial and chloroplast SAG datasets were assessed by exclusion of SAGs contaminated by host, other organelle, and others. **B** Genome coverages in mitochondrial and chloroplast SAGs. Fraction of the reference covered at ≥ 1 × depth for mitochondria and chloroplasts at four target sequencing depths (1 ×, 10 ×, 100 ×, 1000 ×). Box/violin elements indicate median and interquartile range. **C**-**D** Genome-wide coverage profiles. Heatmaps show coverage tracks from a chloroplast (C) and a mitochondrial (D) SAGs at 100 × sequencing depth. Each window shows 500 bp bin. The genome structures of the chloroplast and mitochondrion are shown below. Green indicates the inverted repeat (IR) regions in the chloroplast genome, while red and orange denote the large repeats in the mitochondrial genome. **E** Coverage evenness. Gini-based inequalities of coverage per SAG at 100 × sequencing depth. An independent-samples *t*-test with Welch’s correction revealed a significant difference (*p* = 0.003). **F **Breadth–evenness relation. Relationships between genome coverage and representation evenness (Gini) for chloroplast and mitochondrial SAGs

For the initial analyses, we excluded multi-mapping reads and retained a single primary alignment per read by mapping with Bowtie2 and filtering the BAM files with samtools before further analysis. Libraries were classified as mitochondrial or chloroplast when > 80% of reads within a SAG mapped to the respective organelle genome. Of 384 SAGs processed, 313 passed quality control, yielding 261 mitochondrial and 52 chloroplast SAG datasets (Fig. 2A). Nuclear DNA contamination was extremely low or undetectable in all SAGs, indicating that the DNase I treatment and the NUMT-masking method were effective. Moreover, the difference in the number of SAGs originating from mitochondria and chloroplasts suggests that it reflects the ratio of the two organelles in the leaf cells. Genome coverage breadth (≥ 1 × depth) increased with sequencing depth for both organelles (Fig. 2B). At standard SAG sequencing condition at 100 × sequencing depth, per-library coverage breadth showed medians of 29.6% for mitochondria (*n* = 233) and 37.5% for chloroplasts (*n* = 51), with maxima of 94.4% and 99.9%, respectively. At ultra-deep sequencing condition at 1000 × sequencing depth, per-library coverage breadth showed medians of 59.5% (*n* = 26) for mitochondria and 84.5% for chloroplasts (*n* = 15), with maxima of 97.2% and 100.0%, respectively. These results indicate that even without targeted capture, individual organelles can yield interpretable fractions of their genomes, and near-complete recovery is achievable in favorable cases. In addition, ultra-deep sequencing over 1000 × sequencing depth is a potential approach for improving genome recovery (Fig. 2B), but it reduces the number of SAGs that can be sequenced in parallel.

### Genome coverage patterns in chloroplasts and mitochondria

Genome representation in single-organelle sequencing data showed systematic differences between the two organelle types. Chloroplast SAGs clustered toward higher genome coverages and higher representation evenness (lower Gini, indicates more even coverage distribution), consistent with intact chloroplast genomes within single organelles (Fig. 2B, C, and E). Coverage across chloroplast genomes showed mirrored patterns across inverted repeat (IR) regions (Fig. 2C), consistent with the duplicated structure of these regions. In contrast, mitochondrial SAGs were more dispersed and frequently fragmented, with elevated representation inequality and rugged read-depth profiles (Fig. 2B, D, and E), in line with known structural heterogeneity and coexisting isoforms of plant mtDNA [8]. Notably, it was demonstrated that short regions highly sequenced in multiple SAGs are shared, as seen at positions approximately 50 kb, 195 kb, and 260 kb of mitochondrial SAGs, which correspond to large repeats (LRs) (Fig. 2D). This may reflect preferential amplification, shared rearranged isoforms, or common structural features enriched in the mitochondrial population. The difference between chloroplast IRs and mitochondrial LRs likely reflects repeat architecture and amplification pattern. Chloroplast IRs (approximately 26 kb) are long and highly homologous, which dilutes read assignment [36, 37]. Mitochondrial LRs (approximately 6.5 kb, and approximately 4.2 kb) are much shorter [38]. When we re-performed mapping while retaining multi-mapping reads, coverage depth within the chloroplast IRs increased (Additional file 1: Figure S6A). Mitochondrial coverage showed little change under the same setting (Additional file 1: Figure S6B). This suggests that part of the earlier IR deficit reflected the exclusion of multi-mapping reads, but the slight lower depth remained relative to single-copy regions. In addition, an inverse relation between breadth and Gini coefficients is observed in both organelle types (Fig. 2F), indicating that libraries with broader coverage also show more even read distributions. As a reference, we calculated Gini coefficients for publicly available bulk sequencing datasets of *A. thaliana* that reached 100% organelle genome coverage (SRR32000730, SRR34753010, SRR34753014). The bulk chloroplast libraries showed very low Gini between 0.039 and 0.058, and the bulk mitochondrial libraries showed Gini between 0.19 and 0.32 at full coverage. The higher unevenness of SAG libraries may be a characteristic of single-organelle sequencing, while it may also result from the properties of the amplification bias.

### Characterization of amplification hotspots and aggregate coverage in SAGs

**Fig. 3:**
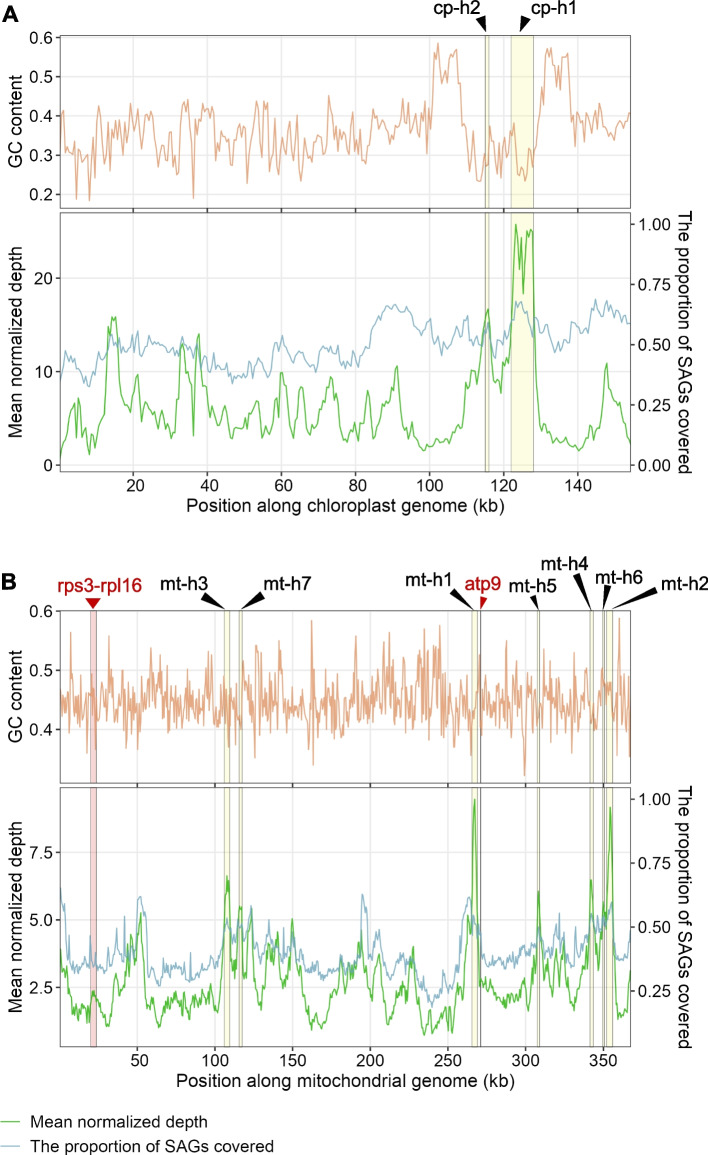
Organellar genome coverage and hotspots across SAGs. **A**-**B** GC content, mean normalized depth, and the proportion of SAGs with coverage along the chloroplast (A) and mitochondrial (B) genomes. Green line shows the mean normalized depth across SAGs; blue line shows the proportion of covered SAGs; orange curve indicates GC content. Metrics were calculated in non-overlapping 500 bp bins. Yellow shaded regions indicate coverage hotspots, defined as regions spanning at least two consecutive 500 bp bins whose mean normalized depth falls within the top 5% of all bins

Direct use of raw read-mapping data can introduce artifacts, particularly for mitochondrial genomes with extensive repeats. Reads may be ambiguously mapped to repeat regions, which can inflate estimates of the number of SAGs carrying these repeats. Therefore, genome coverage patterns were quantified using assembled mitochondrial and chloroplast contigs, and read depth was summarized along these contigs. Each organellar genome was divided into non-overlapping 500 bp bins, and for each bin we calculated two complementary metrics, the mean normalized depth and the proportion of SAGs with coverage. These two measures showed closely matched profiles along both genomes. In mitochondrial SAGs, local peaks in mean depth coincided with peaks in the proportion of covered SAGs, indicating that regions with higher depth tend to be present in a larger fraction of SAGs (Fig. 3B). However, GC content was not clearly associated with either measure, suggesting that the coverage hotspots are not driven by local GC content (Fig. 3).

Moreover, we identified coverage hotspots, defined as genomic regions that contained at least two adjacent 500 bp bins with mean normalized depth in the top 5% of all bins. Two chloroplast coverage hotspots were detected near 115–128 kb (Fig. 3A). These hotspots overlapped the *ccsA* to *ndhD* interval around 115 kb (cp-h2, 115001–116000) and the *ndhA*, *ndhH*, *rps15* and *ycf1* gene cluster close to the small single-copy and IR boundary (cp-h1, 122001–128000). In the mitochondrial SAGs, seven coverage hotspots were also detected (Fig. 3B). mt-h1 (265501–269000 bp) and mt-h5 (307501–309000 bp) overlapped the *atp6* and *ccmC* loci, respectively, and were associated with flanking repeat sequences. mt-h7 (115501–117500) mapped to the *cox1* region, included transcription-related elements, and clustered organellar short RNAs (cosRNAs). The mt-h2 (352001–356000) and mt-h3 (106001–109500) corresponded to regions with dense cosRNA annotations, whereas mt-h4 (341501–343500) and mt-h6 (349501–350500) were located near uncharacterized open reading frames (ORFs). In both chloroplast and mitochondrial hotspots, GC content was not systematically higher or lower than in the rest of the genome, which suggests that the enrichment is not mainly caused by GC-driven sequencing bias. The concurrent increase in both mean normalized depth and SAG-level coverage is instead consistent with local copy-number variation or structural features of these regions, although some bias from WGA cannot be excluded.

### Detection of junction-supporting reads for the *atp9*-*rps3*/*rpl16* rearrangement in mitochondrial SAGs

The mitochondrial SAGs showed diverse coverage patterns. We next asked whether these data could be used to examine structural changes, such as rearrangements, which are frequent and extensive in mitochondrial genomes [39]. As a representative example, we focused on a typical *Arabidopsis* mitochondrial rearrangement involving *atp9* and the *rps3*-*rpl16* region (the *atp9*-*rps3/rpl16* rearrangement [40]; the loci were shown in Fig. 3B).

**Fig. 4:**
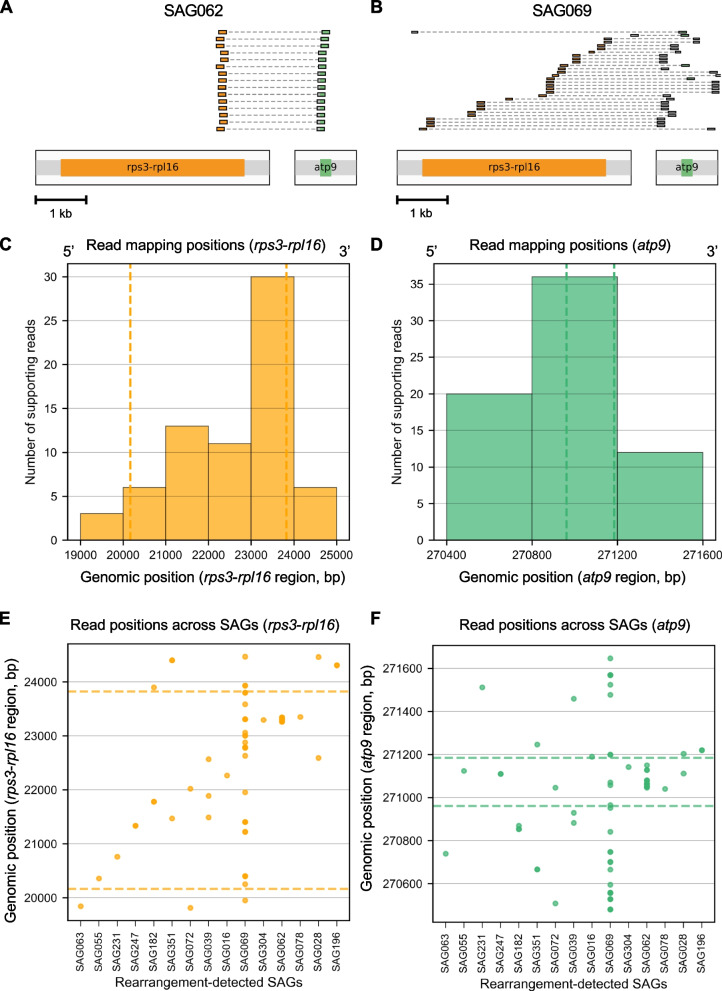
Quantification and distribution of rearrangement junction positions in single mitochondrial genomes. **A**-**B** Representative junction-supporting paired-end read pairs from SAG062 (**A**) and SAG069 (**B**). Each row shows one read pair with mates mapping to the *rps3*-*rpl16* and *atp9* windows. Alignments are drawn to scale; orange and green indicate the *rps3*-*rpl16* and *atp9* gene regions, and gray indicates 500 bp flanking regions. Dashed lines connect read mates. Scale bar, 1 kb. **C**-**D** Distribution of junction-supporting read positions at the *rps3*-*rpl16* locus (**C**) and *atp9* locus (**D**). Histograms show paired-end reads (orange for *rps3*-*rpl16*, green for *atp9*) mapped to each junction position. Dashed vertical lines indicate gene boundaries (*rps3*-*rpl16*: 20,165 and 23,824 bp; *atp9*: 270,961 and 271,185 bp). **E**–**F** Diversity of junction positions across individual rearrangement-positive SAGs at the *rps3*-*rpl16* locus (**E**) and *atp9* locus (**F**). Each dot represents a junction-supporting mate from a single SAG at the indicated genomic position. Dashed vertical lines indicate gene boundaries, and x-axis tick marks denote individual SAGs

To detect this junction in individual SAGs, we searched for paired-end reads in which one mate mapped within a window around the *rps3*-*rpl16* region and the other mapped within a window around *atp9* (using primary alignments with MAPQ ≥ 20). For each SAG, we counted such reads and recorded the positions of the junction-supporting reads at both loci. Junction-supporting reads were detected in multiple mitochondrial SAGs (Fig. 4A-B). Among the 15 SAGs in which we detected the *atp9*-*rps3*/*rpl16* rearrangement, the distribution of junction-supporting read positions on the two sides of the junction showed clear spatial differences (Fig. 4). On the *rps3*-*rpl16* side, supporting positions were dispersed across several kilobases and were enriched toward the 3′ boundary (Fig. 4C and E). On the *atp9* side, supporting positions were highly concentrated near the 5′ boundary, defined here by genomic coordinates rather than transcriptional direction (Fig. 4D and F). These results indicate that the junction-supporting evidence was distributed across multiple positions, rather than being confined to a single coordinate. Notably, we detected multiple distinct junction supporting positions in some SAGs, and SAG069 had the largest number of distinct junction positions (Fig. 4B, E, F). We did not observe a clear relationship between the number of detected junction positions and the total number of mapped reads per SAG (Additional file 2: Table S1). In addition, these patterns were consistent when we relaxed the mapping-quality threshold (MAPQ = 0), suggesting that it is not driven by MAPQ-based filtering (Additional file 1: Figure S7). However, because parts of *rpl16* (and *atp9*) occur as duplicated fragments elsewhere in the reference, local sequence mappability may still influence the apparent positional profile of junction-supporting reads.

On the other hand, we used public PacBio high-fidelity (HiFi) sequencing data for *Arabidopsis* (ERR6210723 [41] and CRR302668 [42]) to place these short-read signals in the context of long-read evidence. We extracted HiFi reads containing sequences from *atp9* and *rpl16*, clustered the extracted reads at 95% identity, and selected the longest read in each cluster as a representative sequence spanning both regions. After we removed clusters with strong nuclear matches by blastn to reduce NUMT like sequences, we obtained three junction patterns (Additional file 1: Figure S8). Paired-end reads from this study supported two of the three junction patterns that were defined from Arabidopsis HiFi long-reads. Pattern A was detected in 37 SAGs, including SAG063, SAG072, SAG078, and SAG182. Pattern B was detected in two SAGs (SAG062 and SAG078). However, Pattern C was not detected, likely because the paired-end library insert size (approximately 300 to 350 bp) was shorter than the span required to capture the diagnostic regions for Pattern C (approximately 700 bp). Together, these results show that single organelle sequencing can capture both local variation in junction supporting read positions within SAGs and broader diversity of junction patterns across SAGs.

## Discussion

To obtain massive SAGs at once, it is desirable to first mitigate organelle genome amplification biases between samples. Methodologically, several extensions follow from our quantitative readouts. When compared with single-cell genome sequencing of eukaryotic cells processed with analogous chemistries and depth [43], our single-organelle libraries show systematically higher mean Gini indicating a more pronounced sequencing bias at the organelle scale. We have recognized that SAG-gel introduces amplification bias on the order of in-tube MDA [44]. However, amplification bias depends on genome size and structure [26, 45], and the same organism can show very different Gini across amplification methods [46]. Thus, our organelle-scale patterns likely reflect both intrinsic genome features and method effects; developing an organelle-tuned amplification approach should be a priority for future work. We therefore prioritize remedies that target representation evenness: targeted capture of low-representation windows, especially for mitochondria, can increase breadth [47]. Optimization of polymerase, primers, and droplet conditions may reduce amplification bias [48–50]. Another approach is to improve upstream purification. While density gradients enrich intact organelles [33, 34, 51], they may leave residues that hinder droplet and WGA. Size- or fluorescence-based sorting (e.g., deterministic lateral displacement, DLD) offers cleaner input but remains challenging for mitochondria [52]. Coupling this workflow with spatial sampling or cell-type enrichment would connect organelle genomes to tissue context. Although full spatial registration lies beyond the scope of this study, the present results provide the necessary per-organelle metrics and throughput to make such studies tractable.

As a potential extension, droplet-based barcoding platforms enable high-throughput barcode assignment and pooled library preparation, and have been successfully applied to recover thousands of individually resolved microbial genomes from complex communities [53–55]. Barcode diversity can be further expanded using split-and-pool strategies, supporting highly multiplexed genome-scale assays [55, 56].

We note limitations. Representation bias at the single-organelle scale remains prominent, and we did not fully disentangle technical from biological contributions; future work will address this with targeted capture, chemistry optimization, and cross-run benchmarking against external references. Finally, while NUMTs masking reduces misassignment, broader evaluation across diverse nuclear backgrounds is needed before application to natural accessions and crops.

Single-organelle genomics enables several applications beyond technical optimization. Previous work has documented mitochondrial heteroplasmy and shifts in mtDNA stoichiometry [57], as well as population-level plastid haplotype divergence from whole-plastome sequencing [58]. However, these studies rely on bulk measurements and therefore do not directly resolve haplotypes at the level of individual organelles. Single-organelle DNA sequencing provides a complementary view. This approach enables haplotype-level readouts from individual organelles and, in principle, quantifies the distribution of organelle variants within a single plant. In this study, we used this approach to explore possible links between structural variation of mtDNA and diversity among mitochondria within cells. We focused on the *atp9*-*rps3*/*rpl16* rearrangement as one example. In mitochondrial SAGs that carried this rearrangement, junction supporting read positions on the two sides of the junction showed different spatial patterns, and some SAGs contained several distinct junction positions. These observations are consistent with models in which plant mitochondrial genomes behave as dynamic populations of recombining subgenomic molecules rather than a single stable structure [8, 57]. At the same time, because the number of informative SAGs is limited and coverage is uneven, we cannot fully distinguish true structural diversity from technical effects such as amplification bias, read mappability, or sampling variation. In addition, public PacBio HiFi long-reads suggest that these loci can produce several major junction patterns, and our paired-end SAG data supported two of these patterns. However, the short insert size of the paired-end libraries in this study limited detection of the full pattern set. In future work, longer insert libraries or long-read sequencing should improve reconstruction of junction diversity and enable more direct assessment of structural heterogeneity at the single-organelle level. Even with current limitations, paired-end single-organelle data can provide junction-supporting evidence that is informative for studying mtDNA structural variation and its diversity across mitochondria.

Structural changes in mtDNA have been proposed to affect mitochondrial function. Rearrangements and copy-number shifts can create or remove chimeric ORFs and alter the genomic context of respiratory genes, which in turn changes mitochondrial function and plant phenotypes [59–61]. Our single-organelle framework provides a basis for future work to connect mtDNA structural heterogeneity to functional variation in mitochondria and to phenotypic outcomes. More broadly, single-organelle genomics should help distinguish coexisting organelle haplotypes within individual plants and track structural heterogeneity across tissues and stress conditions. With further workflow improvements, analogous single-organelle RNA sequencing may also become feasible to assess transcriptional heterogeneity among individual organelles.

## Conclusions

Adapting a droplet workflow to plant organelles enables high-throughput single-organelle DNA-sequencing and resolves organelle-specific representation landscapes: chloroplast genomes are recovered more uniformly within individual organelles, whereas mitochondrial genomes remain more fragmented and heterogeneous. With hundreds of organelle SAGs per run and mapping-based genome coverage breadth routinely reaching tens of percent and occasionally near completeness, this scalable approach provides a practical entry point to dissect recombination, rearrangements, and heteroplasmy across tissues, conditions, and species.

## Materials and methods

### Plant materials

*A. thaliana* Columbia-0 (Col-0) was grown at 22 °C and under long-day conditions (16 h light, 8 h dark). Col-0 seeds were sown on 1/2 Murashige and Skoog (MS) medium (pH5.7) containing 2.3 g/L of MS Plant Salt Mixture (Wako), 500 mg/L of MES, 10 g/L of sucrose, 1 ml/L of Plant Preservative Mixture (Plant Cell Technology), 1 ml/L of Gamborg’s Vitamin Solution (Sigma-Aldrich) and 5 g/L of agar. Seedlings at 2–3 weeks old were transferred to Jiffy-7 (Jiffy Products International) in the greenhouse at 22 °C under long-day conditions. Leaves of 39 DAS (days after stratification) Col-0 were collected and used for organelle isolation.

### The isolation and pretreatment of organelles

Organelle isolation followed previously reported methods [33, 34] with modifications. Leaves were ground in the buffer (300 mM sorbitol, 50 mM HEPES–KOH (pH 7.5), 2 mM MgCl₂, 1 mM EGTA, and 5 mM DTT) using a plastic pestle. The homogenate was centrifuged at 500 × g for 2 min at 4 °C, and the supernatant was collected. The supernatant was sequentially filtered through filter paper, 33 µm and 10 µm strainer (Mini cell strainer, pluriSelect Life Science UG (haftungsb.) & Co.KG) and centrifuged at 1,500 × g for 5 min at 4 °C to collect the organelle-containing supernatant. RNase-Free DNase Set (QIAGEN) was applied to remove free DNA. Invitrogen™ UltraPure™ 0.5 M EDTA (pH 8.0; Thermo Fisher Scientific Inc.) was then added (final concentration of 5 mM) to inhibit DNase, followed by three washes with the buffer to obtain an organelle suspension.

### Massively parallel generation of single-organelle SAGs

SAGs of organelles were obtained using the SAG-gel method [25, 27, 28]. Organelle suspensions were adjusted to 0.3–0.4 organelles/droplet in 1.5% agarose-PBS and encapsulated using an On-chip Droplet Generator (On-chip Biotechnologies Co., Ltd.). After solidification on ice, gel capsules were recovered with 1H, 1H, 2H, 2H-perfluoro-1-octanol (Sigma-Aldrich), washed sequentially with acetone (FUJIFILM Wako Pure Chemical Corporation), isopropanol (FUJIFILM Wako Pure Chemical Corporation), and PBS. Organellar membranes were lysed using chemical lysis with 0.1% (v/v) Triton X-100 (Sigma-Aldrich) on ice for 10 min. After washing and suspending in Buffer D2, whole genome amplification was performed using REPLI-g Single Cell Kit (QIAGEN) at 30 °C for 3 h. Fluorescence-positive gel capsules were identified by 1 × SYBR Green I (Thermo Fisher Scientific) staining and sorted into 384-well plates using FACS (BD Bioscience) for storage at − 30 °C or secondary amplification.

### Library construction and sequencing

SAG libraries were prepared using the QIAseq FX DNA Library Kit (QIAGEN). The ligation adaptors were modified using TruSeq Compatible Full-length Adapters UDI (Integrated DNA Technologies, Inc., IA, United States). Each SAG library was sequenced with the DNBSEQ-G400 2 × 150 bp configuration (MGITech Co., Ltd., Beijing, China) using the MGIEasy Universal Library Conversion Kit.

### Sequencing data processing and mapping

Raw paired-end reads were trimmed for adapters and quality using fastp [62] v0.23.4 with the –detect_adapter_for_pe option and default parameters. The resulting high-quality reads were aligned to the *A. thaliana* reference genome assembly (GCF_000001735.4) using Bowtie2 [63] v2.5.2 with the –very-sensitive preset. To prevent ambiguous mapping to the known NUMTs on chromosome 2 [35], this region was masked with 'N's in the reference genome prior to mapping. The coordinates of the NUMTs region were identified by aligning the mitochondrial genome (BK010421) [64] against the nuclear genome using blastn [65] v2.15.0 (-evalue 1e-4). Mapped reads were downsampled using samtools [66] v1.9 with the view -s option to achieve target mean mapping depths, with sampling fractions calculated from the ratio of target to observed mean mapping depth. For the initial analyses, only primary alignments were kept (samtools view -F 2304). The per-base read depth was calculated from the final BAM file using bedtools [67] v2.31.1 genomecov with the -d option. As an additional analysis, we re-mapped with Bowtie2 (–very-sensitive -k 2), kept multi-mapping alignments with samtools (view -F 2048), and recalculated per-base depth in the same way. This allowed us to assess the effect of excluding versus retaining multi-mapping reads on coverage of chloroplast IRs.

### Coverage evenness (Gini) and visualization

Coverage evenness (Gini) was calculated from per-base read-depth for each SAG extracted from the downsampled coverage summary (100 × sequencing depth). For each SAG the per-base read depth across the organelle reference (including zero-coverage positions) was sorted in ascending order, cumulative sums were computed, and the Gini coefficient was calculated as

$$G=\frac{n+1-2\Sigma \left(cumulative\right)/\Sigma \left(x\right)}{n} \text{ if } \sum \left(x\right)>0,\text{ and } G =0 \text{ if } \sum \left(x\right) =0.$$

where $x$ is per-base read-depth and $n$ is the number of reference positions. Genome coverage breadth was defined as the fraction of reference positions with read-depth ≥ 1. The relationship between Gini (coverage evenness) and genome coverage breadth was assessed by plotting per-SAG Gini against breadth and fitting an ordinary least-squares regression line (lm in R). All calculations and plotting were implemented using custom Python (pandas/numpy) and R (tidyverse/ggplot2) scripts.

### Identification of mitochondrial and chloroplast coverage hotspots across SAGs

To reduce bias from direct mapping of raw reads to repeat regions, mitochondrial genome coverage patterns were quantified using assembled mitochondrial contigs from low-contamination SAGs. These contigs were aligned to the reference mitochondrial genome (BK010421) using minimap2 v2.26 with the asm5 preset for assembly-to-reference alignment (-ax asm5 -t 2). Alignments were converted to sorted BAM format using samtools v1.9, and per-base coverage depth was extracted for each SAG. To account for sequencing depth variation among SAGs, total-depth normalization was implemented by calculating the sum of coverage across all mitochondrial positions for each SAG, then normalizing each position's depth as (depth/total_depth_per_SAG) × 1 × 10⁶. The normalized per-base coverage data were aggregated into 500 bp bins, and for each bin the mean normalized depth across all SAGs and the proportion of SAGs with detectable coverage were calculated. Peak regions were identified as bins with mean normalized depth in the top 5% genome-wide, excluding isolated single-bin peaks and retaining only contiguous regions spanning ≥ 2 consecutive 500 bp bins. Coverage distributions and peak regions were visualized using circular genome plots generated with custom R (data.table/ggplot2/patchwork) scripts. Chloroplast SAGs were processed using identical data processing, while the IR-B region was excluded from the chloroplast reference genome prior to alignment.

### Analysis of *atp9*-*rps3*/*rpl16* rearrangement in mitochondrial SAGs

#### Direct mapping analysis based on short reads

To quantify mitochondrial genomic rearrangements, we first remapped the fastp filtered paired-end reads to the NUMTs-masked A. thaliana reference genome. We used BWA MEM v0.7.17 with default settings and then sorted the alignments by coordinate using samtools v1.9. For each single organelle library we retained primary and supplementary alignments in per sample BAM files. A custom Python pipeline was then developed using pysam v0.22.0 to parse these BAM alignments. The analysis targeted junctions connecting the *rps3*-*rpl16* gene cluster (treated as the left junction region, reference coordinates 20,165–23,824) and the *atp9* locus (treated as the right junction region, reference coordinates 270,961–271,185). Windows encompassing the full gene regions were extended by 500 bp on both sides to capture junction-spanning reads. Rearrangement-supporting evidence was identified from discordant paired-end reads whose two mates mapped to the respective *rps3*-*rpl16* and *atp9* windows. Only high-confidence alignments (–min-mapq 20) from regions with sufficient coverage (–min-coverage 5) were considered. Junction-supporting read positions from multiple reads within each sample were clustered using a 50 bp tolerance window to define consensus junction positions. All junction-supporting reads were further checked for non-specific alignments outside the defined target windows. The junction-supporting reads with any alignment record mapping externally were excluded to maintain stringent specificity. Finally, the distribution and inter-sample variability of these junction positions were quantified and visualized using Python (pandas/matplotlib).

#### Short read mapping guided by junction patterns defined from public PacBio HiFi sequencing data

Public Arabidopsis HiFi long-read data (ERR6210723 [41] and CRR302668 [42]) were analyzed to infer junction patterns between *atp9* and the *rps3*-*rpl16* region. Reference sequences consisting of *atp9* with 500 bp upstream and downstream flanks and *rps3*-*rpl16* with 500 bp upstream and downstream flanks were constructed, and HiFi reads were mapped to these references. Reads that contained both loci within a single read were retained and clustered using CD-HIT-EST v4.8.1 [68] (-c 0.95 -n 10 -aS 0.9 -d 0 -g 1). The representative reads for each cluster were queried against the Arabidopsis nuclear genome using blastn, and clusters were removed when the top nuclear hit covered 90% or more of the read length to reduce NUMT like sequences. From each retained read, the segment spanning *atp9* plus 1 kbp upstream and 1 kbp downstream was extracted. Extracted sequences were clustered at 95% sequence identity using CD-HIT-EST (-c 0.95 -n 10 -d 0 -g 1). Clusters were used to define three junction patterns and to construct pattern reference sequences. Paired-end short reads generated in this study were quality filtered with fastp v0.23.4 using –detect_adapter_for_pe and -w 1. Filtered reads were competitively mapped to the pattern references with minimap2 v2.26 using -ax sr -t 1, and alignments were converted to BAM and name sorted with samtools v1.9. Read pairs were required to be proper pairs and to map to the same pattern reference. Coordinate based criteria were applied with an awk script that computed reference end positions from CIGAR strings. For Pattern A, one mate had to span position 200 and the other mate had to span position 745. For Pattern B, one mate had to overlap 126 to 162 and the other mate had to overlap 489 to 707. For Pattern C, one mate had to extend to at least position 746 and the other mate had to overlap 201 to 263. Each read name was counted at most once per pattern. All positions reported above refer to coordinates on the pattern reference sequences.

### Quantitative PCR (qPCR) analysis

DNA based qPCR on crude organelle extracts before and after DNase I treatment. DNA was purified from the organelle suspensions prepared as described above using Template Prepper for DNA (NIPPON GENE CO., LTD.) according to the manufacturer’s instructions.

One nuclear locus (*cyo1*) and two mitochondrial loci (*cox1* and *rrn26*) were quantified. The qPCR analysis was conducted using GeneAce SYBR qPCR Mix α Low ROX (NIPPON GENE CO., LTD.) on a StepOnePlus Real-Time PCR System (Applied Biosystems). The reaction volume (20 μL) contained 0.2 μM of each primer and 2 μL of DNA template. The mitochondrial primer pairs were designed in regions that do not overlap the known NUMT insertion on chromosome 2. Four biological replicates were prepared and analyzed. Nuclear DNA depletion was evaluated from the increase in *cyo1* Ct after DNase I treatment. Relative mtDNA abundance was calculated by the ΔΔCt method with *cyo1* as the reference locus (crude sample as reference sample). These data were used to compare nuclear and mtDNA levels in organelle preparations and to assess the potential contribution of NUMTs to mitochondrial read assignment. The primers used for qPCR were designed for *A. thaliana* Col-0 genes as follows:

- *cyo1* forward, 5′-GGAGGTTGCTCTGGTCGAAA-3′ and reverse, 5′-ATCACATTGCAACCTCCGGT-3′.
- *cox1* forward, 5′-TTCAGGGTATGTCCGACCAAAG-3′ and reverse, 5′- TGGCAAATTCAGGGCTAGACAT-3′.
- *rrn26* forward, 5′-CTCCAAGAGAGAGGCATGGTTT-3′ and reverse, 5′-AGAGTGAATCGGTCCCTAAGGA-3′.

### PCR amplification and gel electrophoresis

The PCR reaction mixture consisted of the following: 5 μl KAPA HiFi HotStart ReadyMix (NIPPON Genetics Co, Ltd.), 2 μL sterile distilled water (SDW), 1 μL of each primer (2 μM forward and reverse primer), 1 μL crude preparation (50-fold dilution). The PCR reaction was performed using T100 Thermal Cycler (Bio-Rad Laboratories, Inc.) with the following program: the initial denaturation step at 95 °C for 3 min, then followed by 36 cycles of denaturation at 95 °C for 15 s, annealing at 57 °C for 15 s, and extension at 72 °C for 15 s. This was followed by a final extension step at 72 °C for 30 s. PCR products were mixed with 6 × loading buffer (Takara Bio Inc.) and Midori Green Direct (NIPPON Genetics Co, Ltd.) at the recommended ratio and electrophoresed on a 2% agarose gel at 100 V for 25 min. Invitrogen™ 1 Kb Plus DNA Ladder (Thermo Fisher Scientific Inc.) was used as the molecular marker. The primers used for PCR were designed for *A*. *thaliana* Col-0 genes [69] as follows:

- *atp6-2* forward, 5′-CATTCCCGGAAAGACCACCT-3′ and reverse, 5′-CGGGAGCAAACTGGACCTTA-3′;
- *16S rRNA* forward, 5′-GGAGCGGTGAAATGCGTAGA-3′ and reverse, 5′-AAGGTAACGACTTCGGGCAT-3′;
- *cyo1* forward, 5′-CTCGTCTTCCTTCTCGCTCC-3′ and reverse, 5′-TCAAACAGCCAATCGTCAGC-3′.

### Statistical analyses

Statistical analyses were performed using IBM SPSS Statistics (version 28.0). Independent samples *t*-tests were used to compare means between two groups. Homogeneity of variances was examined using Levene’s test. When Levene’s test was not significant (*p* ≥ 0.05), we used the *t*-test that assumes equal variances. When Levene’s test was significant (*p* < 0.05), we used Welch’s *t*-test, which does not assume equal variances. All tests were two-sided, and *p* values less than 0.05 were considered statistically significant.

## Supplementary Information

Additional file 1: Figure S1 PCR detection of organelle and nuclear DNA in crude organelle preparation. PCR was performed using primers targeting (a) the mitochondrial atp6-2 gene, (b) the chloroplast 16S rRNA gene, and (c) the nuclear cyo1 gene in A. thaliana Col-0. Genomic DNA from A. thaliana (AtDNA) was used as a positive control. Amplicons were visualized by agarose gel electrophoresis. Lane M: DNA ladder. Figure S2 Full-length, unprocessed gel image corresponding to Supplementary Figure S1. A, the image used for Supplementary Figure S1. B, the image represents one replicate experiment. (P) Positive control. (T) Crude preparation treated with DNase I. (a) the mitochondrial atp6-2 gene, (b) the chloroplast 16S rRNA gene, and (c) the nuclear cyo1 gene in A. thaliana. Lane M: DNA ladder. Genomic DNA from A. thaliana (AtDNA) was used as a positive control. The gel was trimmed according to the number of samples and to fit the gel apparatus before electrophoresis. No digital cropping or image adjustment was applied after imaging. Figure S3 Extraction buffer affects SAG library composition. A, Representative field of the crude preparation isolated with PBS or sucrose buffer. Chloroplasts appeared fragmented, with numerous cellular debris and impurities. Scale bar: 30 µm. B, PCR detection of organelle and nuclear DNA in crude organelle preparation prepared in PBS. (a) the mitochondrial atp6-2 gene, (b) the chloroplast 16S rRNA gene, and (c) the nuclear cyo1 gene in A. thaliana. Lane M: DNA ladder. C, Library composition and quality control from each buffer condition. Both buffer conditions resulted in severe contamination with Arabidopsis nuclear DNA and low mitochondrial read abundance. Figure S4 Full-length, unprocessed gel image corresponding to Supplementary Figure S3B. PCR was performed using the crude organelle preparation in PBS as the template. (a) the mitochondrial atp6-2 gene, (b) the chloroplast 16S rRNA gene, and (c) the nuclear cyo1 gene in A. thaliana. Lane M: DNA ladder. Only the lanes indicated by red text were used and presented in this study. The other lanes are not described and do not affect the results. The gel was trimmed according to the number of samples and to fit the gel apparatus before electrophoresis. No digital cropping or image adjustment was applied after imaging. Figure S5 Quantitative PCR assessment of relative mtDNA abundance after DNase I treatment. A, ΔCt values for mitochondrial loci cox1 and rrn26 relative to the nuclear locus cyo1 in crude organelle suspensions and DNase I treated suspensions. B, Relative mtDNA abundance for cox1 and rrn26, calculated by the ΔΔCt method with cyo1 as the reference locus and the crude sample as the reference sample. Bars represent the mean ± SD of four biological replicates. Asterisks indicate significant differences between crude and DNase I treated samples (*, p < 0.01; ***, p < 0.001). Figure S6 Genome-wide coverage profiles with multi-mapping reads retained. A-B, Heatmaps show coverage tracks from a chloroplast (A) and a mitochondrial (B) SAGs at 100× sequencing depth. Each window shows 500 bp bin. Figure S7 Quantification and distribution of rearrangement junction positions in single mitochondrial genomes with relaxed mapping-quality filtering (MAPQ = 0). A-B, Distribution of junction-supporting read positions at the rps3-rpl16 locus (A, left) and atp9 locus (B, right). Histograms show paired-end reads (orange for rps3-rpl16, green for atp9) mapped to each junction position.Dashed lines indicate gene boundaries (rps3-rpl16: 20,165 and 23,824 bp; atp9: 270,961 and 271,185 bp). C-D, Diversity of junction positions across individual rearrangement-detected SAGs at the rps3-rpl16 locus (C, left) and atp9 locus (D, right). Each dot represents an individual supporting read within a single SAG. Dashed lines indicate gene boundaries. X-axis tick marks denote individual SAG samples. Figure S8 Schematic of three junction patterns inferred from PacBio high-fidelity (HiFi) sequencing data. Patterns A, B, and C were inferred from Arabidopsis PacBio HiFi sequencing data by clustering reads at 95% sequence identity. Colored boxes indicate flanking segments, gene segments, and spacer segments, and lengths (bp) are shown inside each box. Spacer part 2 corresponds to a partial rps3 sequence. The black arrows indicate the detection conditions for ligation-cross reads used in pattern definition and subsequent classification based on paired-end sequencing.

Additional file 2: Table S1. Total mapped reads and paired-end support for detected atp9-rps3/rpl16 rearrangements. List of SAGs containing detectable rearrangements connecting the atp9 and rps3-rpl16 loci. Total mapped reads reflect total mapped reads, while paired-end counts indicate reads spanning the rearrangement junction.

## Data Availability

Sequencing data have been deposited in the NCBI database under BioProject PRJNA1311696.
