# Supplementary material for: Single-organelle DNA-sequencing of chloroplasts and mitochondria in Arabidopsis thaliana
Source: BMC Plant Biol. 2026 Jan 26;26:335. doi: 10.1186/s12870-026-08232-3 (PMC12914924; doi:10.1186/s12870-026-08232-3)
Supplement: Supplementary file 1 — Additional file 1: Figure S1 PCR detection of organelle and nuclear DNA in crude organelle preparation. PCR was performed using primers targeting (a) the mitochondrial atp6-2 gene, (b) the chloroplast 16S rRNA gene, and (c) the nuclear cyo1 gene in A. thaliana Col-0. Genomic DNA from A. thaliana (AtDNA) was used as a positive control. Amplicons were visualized by agarose gel electrophoresis. Lane M: DNA ladder. Figure S2 Full-length, unprocessed gel image corresponding to Supplementary Figure S1. A, the image used for Supplementary Figure S1. B, the image represents one replicate experiment. (P) Positive control. (T) Crude preparation treated with DNase I. (a) the mitochondrial atp6-2 gene, (b) the chloroplast 16S rRNA gene, and (c) the nuclear cyo1 gene in A. thaliana. Lane M: DNA ladder. Genomic DNA from A. thaliana (AtDNA) was used as a positive control. The gel was trimmed according to the number of samples and to fit the gel apparatus before electrophoresis. No digital cropping or image adjustment was applied after imaging. Figure S3 Extraction buffer affects SAG library composition. A, Representative field of the crude preparation isolated with PBS or sucrose buffer. Chloroplasts appeared fragmented, with numerous cellular debris and impurities. Scale bar: 30 µm. B, PCR detection of organelle and nuclear DNA in crude organelle preparation prepared in PBS. (a) the mitochondrial atp6-2 gene, (b) the chloroplast 16S rRNA gene, and (c) the nuclear cyo1 gene in A. thaliana. Lane M: DNA ladder. C, Library composition and quality control from each buffer condition. Both buffer conditions resulted in severe contamination with Arabidopsis nuclear DNA and low mitochondrial read abundance. Figure S4 Full-length, unprocessed gel image corresponding to Supplementary Figure S3B. PCR was performed using the crude organelle preparation in PBS as the template. (a) the mitochondrial atp6-2 gene, (b) the chloroplast 16S rRNA gene, and (c) the nuclear cyo1 gene in A. thaliana. La [file 12870_2026_8232_MOESM1_ESM.docx]

Supplemental information for:

**Single-organelle DNA-sequencing of chloroplasts and mitochondria in Arabidopsis thaliana**

**Zikai Xiang^1^, Kazuki Takahashi^2,5^, Chang Zhou**^3^**, Hideki Takanashi**^3^**, Shin-ichi Arimura**^3^**, Masahito Hosokawa^1,2,4*^**

^1^ Waseda Research Institute for Science and Engineering, Waseda University, 3-4-1 Okubo, Shinjuku-ku, Tokyo 169-8555, Japan

^2^ Research Organization for Nano and Life Innovation, Waseda University, 513 Wasedatsurumaki-cho, Shinjuku-ku, Tokyo 162-0041, Japan.

^3^ Graduate School of Agricultural and Life Sciences, The University of Tokyo, 1-1-1 Yayoi, Bunkyo-ku, Tokyo, 113-8657, Japan

^4^ Graduate School of Advanced Science and Engineering, Waseda University, 2-2 Wakamatsu-cho, Shinjuku-ku, Tokyo 162-8480, Japan.

^5^ Microbe Division/Japan Collection of Microorganisms, RIKEN BioResource Research Center, Tsukuba, Ibaraki 305-0074, Japan.

* Correspondence: Masahito Hosokawa [masahosokawa@aoni.waseda.jp](mailto:masahosokawa@aoni.waseda.jp)

## **SUPPLEMENTARY FIGURES**

**Figure S1 PCR detection of organelle and nuclear DNA in crude organelle preparation.**

PCR was performed using primers targeting (a) the mitochondrial *atp6-2* gene, (b) the chloroplast *16S rRNA* gene, and (c) the nuclear *cyo1* gene in *A. thaliana*. Genomic DNA from *A. thaliana* (AtDNA) was used as a positive control. Amplicons were visualized by agarose gel electrophoresis. Lane M: DNA ladder.


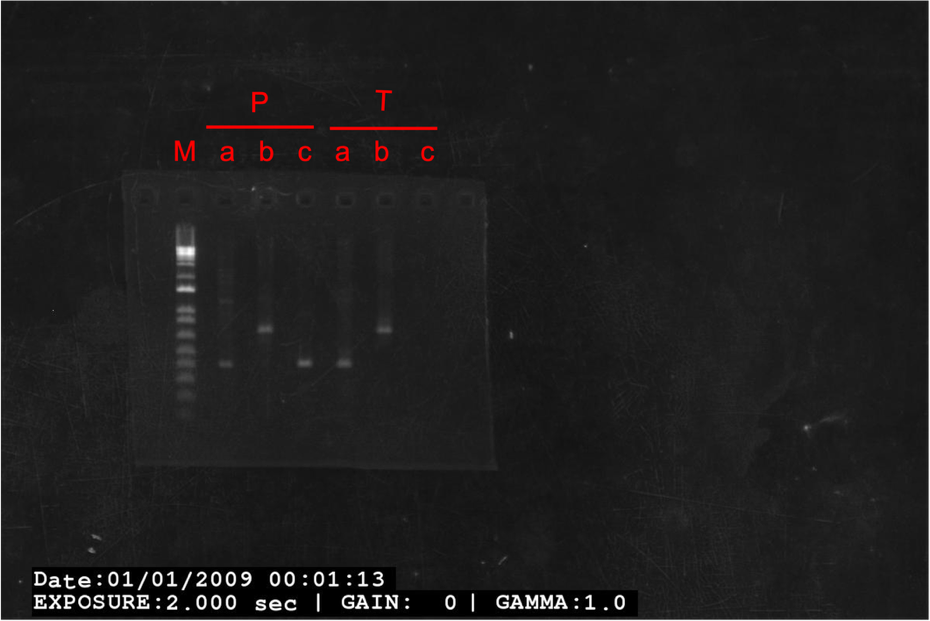
**A**


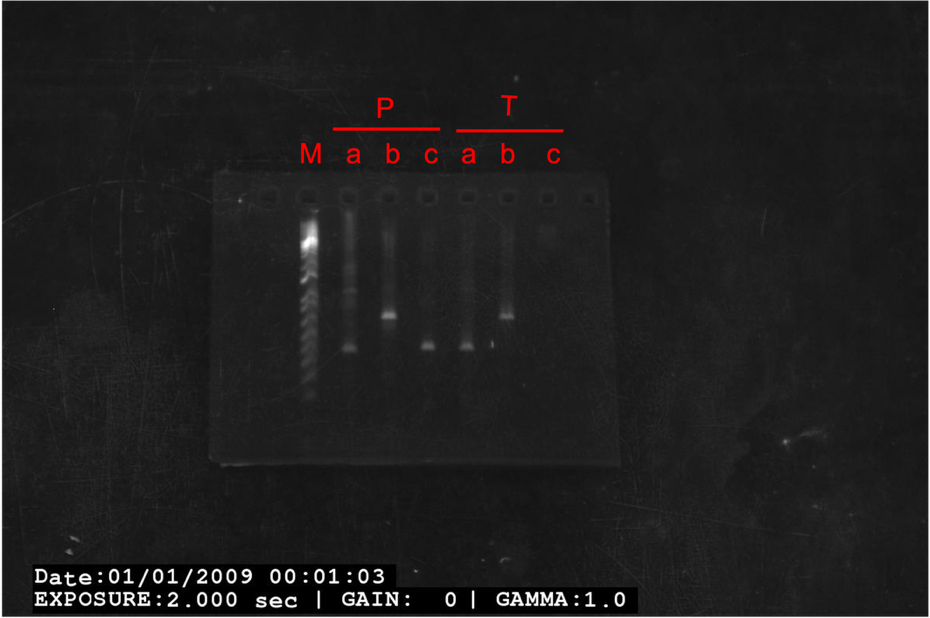
**B**

**Figure S2 Full-length, unprocessed gel image corresponding to Supplementary Figure S1.**

**A**, the image used for Supplementary Figure S1. **B**, the image represents one replicate experiment. (P) Positive control. (T) Crude preparation treated with DNase I. (a) the mitochondrial *atp6-2* gene, (b) the chloroplast *16S rRNA* gene, and (c) the nuclear *cyo1* gene in *A. thaliana*. Lane M: DNA ladder. Genomic DNA from *A. thaliana* (AtDNA) was used as a positive control. The gel was trimmed according to the number of samples and to fit the gel apparatus before electrophoresis. No digital cropping or image adjustment was applied after imaging.

**Figure S3** **Extraction buffer affects SAG library composition.**

**A, Representative field of the crude preparation isolated with PBS or sucrose buffer**. Chloroplasts appeared fragmented, with numerous cellular debris and impurities. Scale bar: 30 µm.

**B, PCR detection of organelle and nuclear DNA in crude organelle preparation prepared in PBS.** (a) the mitochondrial *atp6-2* gene, (b) the chloroplast *16S rRNA* gene, and (c) the nuclear *cyo1* gene in *A. thaliana*. Lane M: DNA ladder.

**C, Library composition and quality control from each buffer condition.** Both buffer conditions resulted in severe contamination with *Arabidopsis* nuclear DNA and low mitochondrial read abundance.


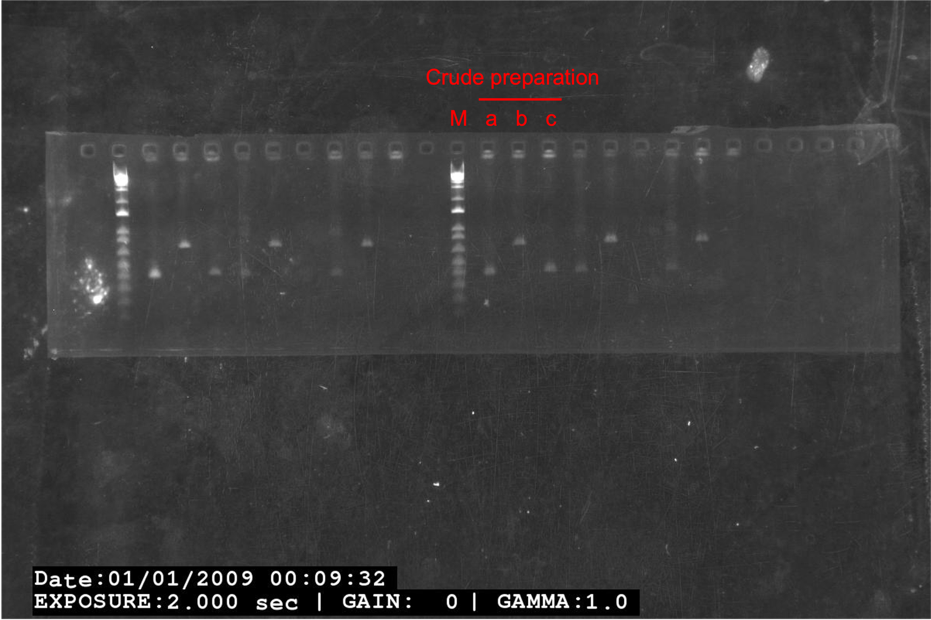


**Figure S4 Full-length, unprocessed gel image corresponding to Supplementary Figure S3B.**

PCR was performed using the crude organelle preparation in PBS as the template. (a) the mitochondrial *atp6-2* gene, (b) the chloroplast *16S rRNA* gene, and (c) the nuclear *cyo1* gene in *A. thaliana*. Lane M: DNA ladder. Only the lanes indicated by red text were used and presented in this study. The other lanes are not described and do not affect the results. The gel was trimmed according to the number of samples and to fit the gel apparatus before electrophoresis. No digital cropping or image adjustment was applied after imaging.


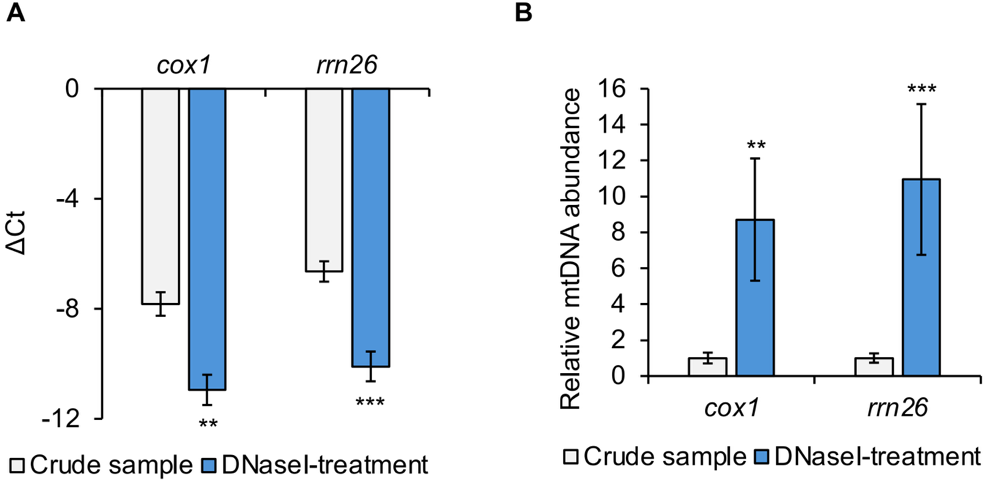


**Figure S5 Quantitative PCR assessment of relative mtDNA abundance after DNase I treatment.**

**A**, ΔCt values for mitochondrial loci cox1 and rrn26 relative to the nuclear locus cyo1 in crude organelle suspensions and DNase I treated suspensions.

**B**, Relative mtDNA abundance for cox1 and rrn26, calculated by the ΔΔCt method with cyo1 as the reference locus and the crude sample as the reference sample. Bars represent the mean ± SD of four biological replicates. Asterisks indicate significant differences between crude and DNase I treated samples (*, p < 0.01; ***, p < 0.001)


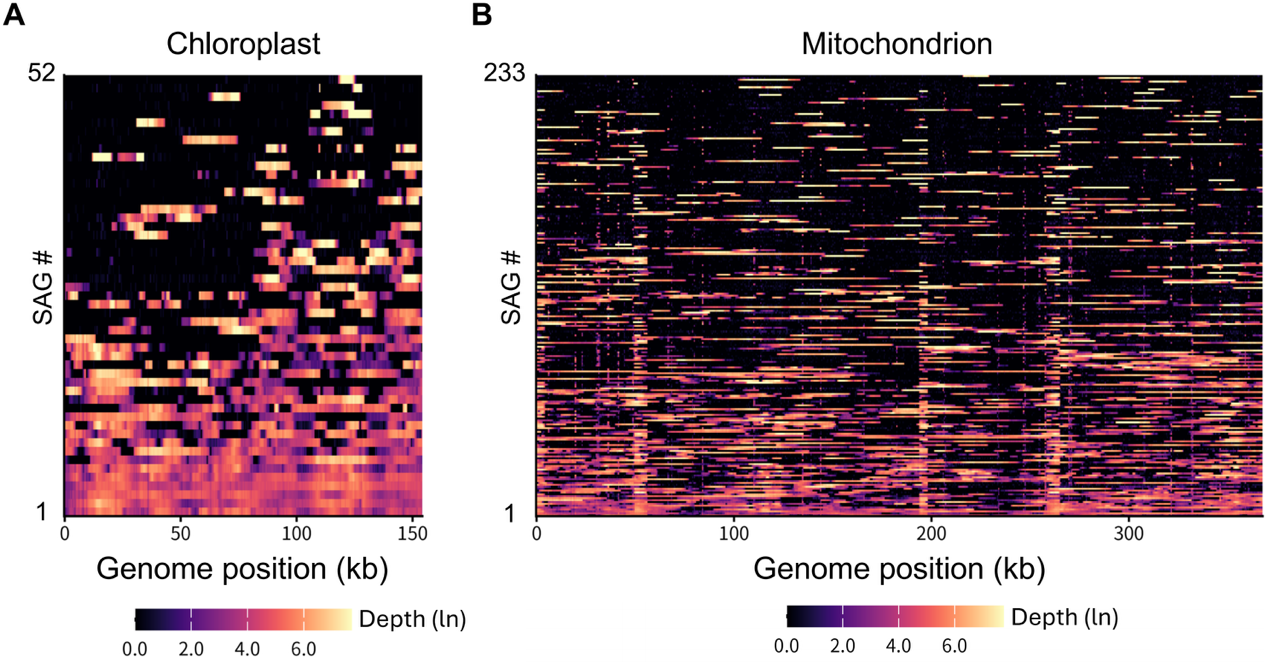


**Figure S6 Genome-wide coverage profiles with multi-mapping reads retained.**

**A-B**, Heatmaps show coverage tracks from a chloroplast (A) and a mitochondrial (B) SAGs at 100× sequencing depth. Each window shows 500 bp bin.

**
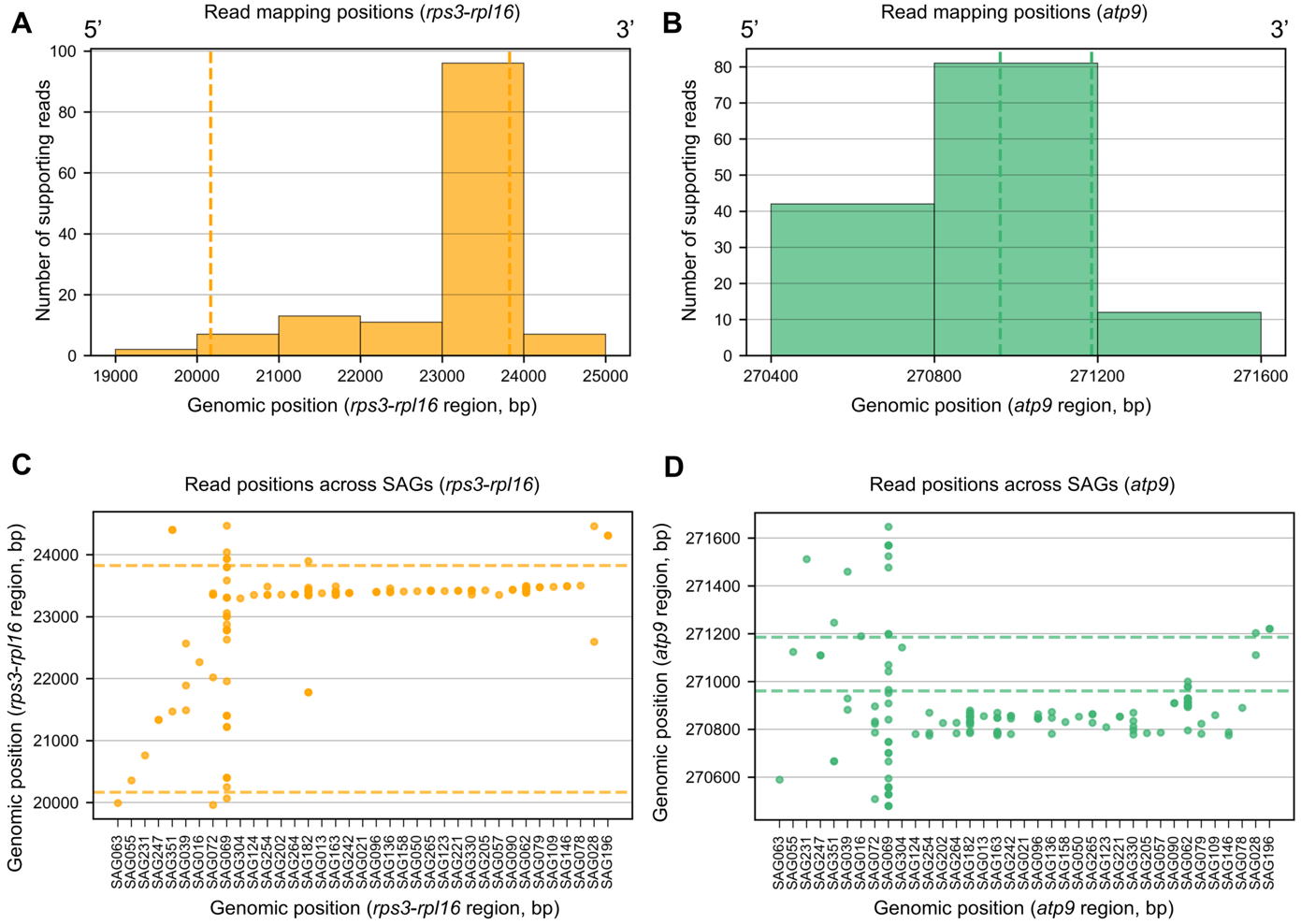
**

**Figure S7 Quantification and distribution of rearrangement junction positions in single mitochondrial genomes with relaxed mapping-quality filtering (MAPQ = 0).**

**A-B**, Distribution of junction-supporting read positions at the rps3-rpl16 locus (A, left) and atp9 locus (B, right). Histograms show paired-end reads (orange for rps3-rpl16, green for atp9) mapped to each junction position.Dashed lines indicate gene boundaries (rps3-rpl16: 20,165 and 23,824 bp; atp9: 270,961 and 271,185 bp).

**C-D**, Diversity of junction positions across individual rearrangement-detected SAGs at the rps3-rpl16 locus (C, left) and atp9 locus (D, right). Each dot represents an individual supporting read within a single SAG. Dashed lines indicate gene boundaries. X-axis tick marks denote individual SAG samples.

**Figure S8 Schematic of three junction patterns inferred from PacBio high-fidelity (HiFi) sequencing data.**

Patterns A, B, and C were inferred from Arabidopsis PacBio HiFi sequencing data by clustering reads at 95% sequence identity. Colored boxes indicate flanking segments, gene segments, and spacer segments, and lengths (bp) are shown inside each box. Spacer part 2 corresponds to a partial rps3 sequence. The black arrows indicate the detection conditions for ligation-cross reads used in pattern definition and subsequent classification based on paired-end sequencing.
